# Aneurysm Specific Working Projections for the Endovascular Treatment of Intracranial Aneurysms—Same Aneurysm, Different Working Projections?”

**DOI:** 10.1007/s00270-025-04236-y

**Published:** 2025-10-16

**Authors:** Roland Schwab, Stefan Klebingat, Elie Diamandis, Harald Paukisch, Sebastian Müller, Eya Khadhraoui, Erelle Fuchs, Hannes Nordmeyer, Christina Wendl, Donald Lobsien, Maximilian Thormann, Frans Van den Bergh, Uta Hanning, Daniel Behme

**Affiliations:** 1 University Clinic for Neuroradiology, University Hospital Magdeburg, Magdeburg, Germany; 2 Department of Neuroradiology Städtisches Klinikum Solingen, Solingen, Germany; 3 Medical School, Department of Health, Witten/Herdecke University, Witten, Germany; 4 Institute of Radiology, University Hospital Regensburg, Regensburg, Germany; 5 Klinikum Chemnitz gGmbH, Institut Für Radiologie Und Neuroradiologie, Chemnitz, Germany; 6 Department of Nuclear Medicine, Charité Berlin, Berlin, Germany; 7 Department of Radiology, Universitair Ziekenhuis Brussel, Vrije Universiteit Brussel (VUB), Brussels, Belgium; 8 Department of Diagnostic and Interventional Neuroradiology, University Medical Center Hamburg-Eppendorf, Hamburg, Germany; 9 Research Campus STIMULATE, Otto-Von-Guericke University Magdeburg, Magdeburg, Germany

**Keywords:** Aneurysm, EVT, Projection, DSA

## Abstract

**Purpose:**

Despite decades of endovascular treatment (EVT) of intracranial aneurysms (IA), there are no studies regarding the ideal working projections. Possible correlations between suboptimal working projections and procedure-related complications therefore remain unknown. This study aims at investigating the level of consensus between different physicians proposed working projections for endosaccular treatment of IAs.

**Materials and Methods:**

Five interventional neuroradiologists used a simulation software to select what they considered to be the optimal biplane working projections (BPW) for the intrasaccular treatment of 20 intracranial aneurysms. Five further raters evaluated these projections and either agreed or disagreed with the proposed working projection. Where necessary, reasons for rejecting the proposed working projection was provided.

**Results:**

Overall, a substantial interobserver consensus was achieved (*κ* = 0.81), with at least one projection consistently agreed upon for 19 out of 20 aneurysms. Conversely, only one projection was unanimously rejected. Lack of clear delineation of the aneurysm neck, the parent vessel, and associated vessels were the most frequently cited reasons for rejection (39%, 39%, and 31%, respectively), while “other reasons” accounted for 19%.

**Conclusion:**

Although the specific working projections chosen for individual aneurysms were heterogeneous, there was still a broad consensus on at least one suitable projection for almost every case. Further research is required to explore the possible benefit of adjusting the patient’s head position or making slight angle deviations in BWPs with physical restrictions and furthermore, to evaluate the risks associated with suboptimal BWPs in EVT.

**Graphical abstract:**

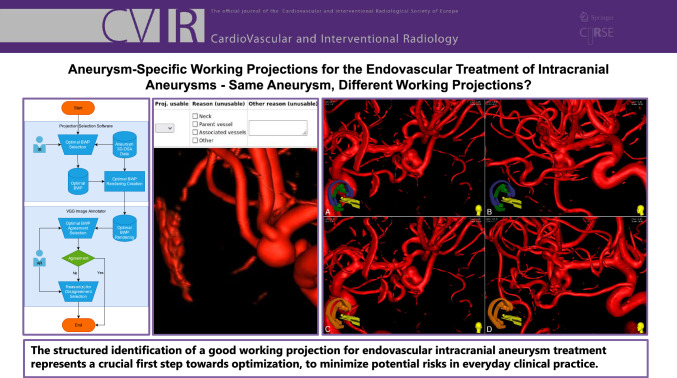

## Introduction

With the increasing number of cross-sectional brain imaging studies, the detection rate of unruptured intracranial aneurysms (UIAs) is rising. The prevalence of UIAs is estimated to be 2–3%. Annually, nearly 500,000 individuals suffer from acute subarachnoid hemorrhage (aSAH), with over 80% of cases being attributed to ruptured intracranial aneurysms (RIAs) [1]. The majority (80–90%) of these intracranial aneurysms (IA) are located in the anterior circulation [2]. For both preventive treatment of UIAs and acute intervention for RIAs, endovascular embolization and microsurgical clipping are the primary therapeutic options. Recent advancements in devices and techniques demonstrate the safety, efficacy, and favorable clinical outcomes of endovascular embolization. These improvements have shifted the preference toward endovascular treatments (EVT) [3–8]. Successful endovascular treatment requires precise delineation of the aneurysm to ensure complete occlusion and to minimize complications. Three-dimensional rotational digital subtraction angiography (3D DSA) with volume rendering (VR) has become the superior imaging modality for treatment planning [9–13]. Accurate visualization of the aneurysm neck, dome, and associated vessels is vital for successful endovascular treatment [10, 13]. In clinical practice, achieving an optimal projection for treatment can be challenging due to C-arm movement constraints or the anatomical location of the aneurysm. These restrictions may limit EVT or increase the risk of complications significantly. However, the process of identifying the optimal working projection has not been the focus of research in the past.

Therefore, this study aims at investigating the level of consensus between different interventional neuroradiologists (INR) proposed working projections for endosaccular treatment of IAs, providing preliminary data to guide future research on these questions and to facilitate the development of an artificial intelligence-assisted application supporting the optimization of working projections.

## Materials and Methods

### Case Selection

We searched our database for intracranial saccular sidewall or bifurcation aneurysms of the anterior circulation with a narrow neck (neck width < 4 mm; dome to neck ratio > 2) from the last 3 years, which from an anatomical standpoint, seemed feasible for coil embolization or intrasaccular flow diversion [14]. This resulted in a total of 207 aneurysms with available 3D DSA imaging of the untreated aneurysm. Overall, we randomly picked 20 aneurysms. Ten aneurysms were treated endovascularly and 10 that were treated via microsurgical clipping. All selected aneurysms had been previously discussed in an interdisciplinary board and were considered amenable to both treatment modalities. The final therapeutic approach was determined by patient preference rather than by technical limitations. The aneurysm characteristics are shown in Table 1.

**Table 1:** Aneurysm characteristics

Characteristics	Values
Size (mm) (mean ± *SD* [range])	6.5 ± 2.3 (3.9–13)
Neck width (mm) (mean ± *SD* [range])	3 ± 0.8 (1.6–4.0)
Dome-Neck ratio (mean ± *SD* [range])	2.2 ± 0.3 (1.4–3.3)
Site (n [%])	–
right	11 (55)
left	9 (45)
Location (n [%])	–
Bifurcation	12 (60)
Sidewall	8 (40)
Topography (n [%])	–
ICA	1 (5)
MCA Bifurcation	8 (40)
ACA (AcomA Complex)	11 (55)

*MCA* Middle cerebral artery, *ACA* Anterior cerebral artery, *AcomA* Anterior communicating artery

### 3D Digital Subtraction Angiography

3D DSA images were acquired with a biplane angiographic unit (Artis Q, Siemens Healthineers GmbH, Erlangen, Germany) in the context of clinical diagnostics, therapy planning or therapy. The isotropic edge length of the voxels is 0.28 mm. The reconstructed volume has a width and height of 142.91 mm each at a uniform resolution of 512 voxels in x- and y-direction. The median depth is 189 voxels (IQR, 169–215) and 52.75 mm (IQR, 47.2–59.7), respectively.

A point of interest (POI) was placed at the approximate center of the aneurysm neck for each target aneurysm. The POI was used to identify the aneurysm uniquely and to provide a consistent visual reference point across different views, as shown in Fig. 4.

### Raters

**Fig. 1:**
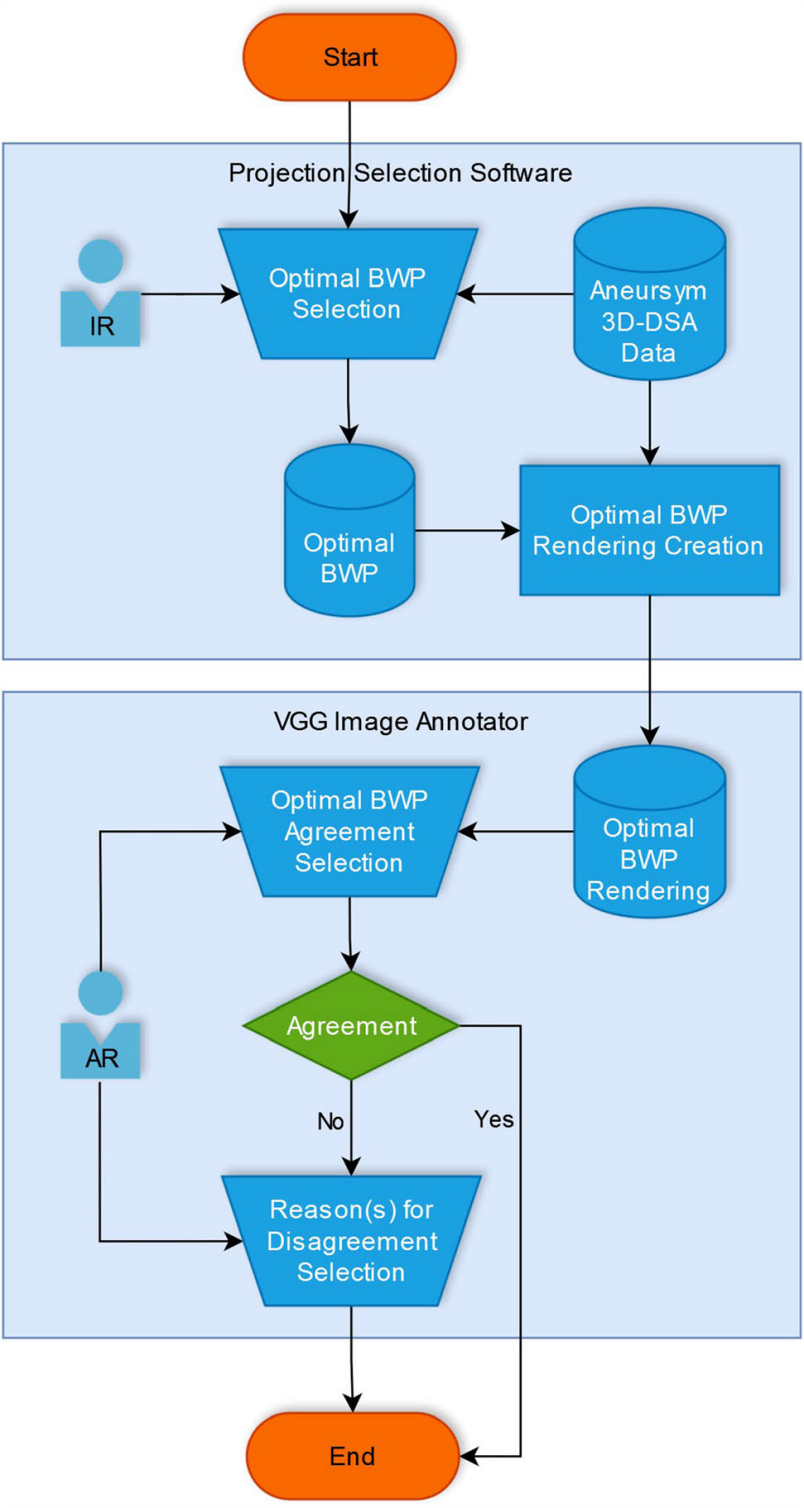
Workflow of the rating process

All images were rated by ten interventional neuroradiologists (INR) from eight different centers. Eight of them having at least ten years of experience in interventional neuroradiology and two of them having more than five years of experience. Initial raters (IR) 1–5 created preferred biplane work projections (BWP). Afterward, agreement raters (AR) 6–10 agreed with or disagreed with the proposed projections. The whole workflow is shown in Fig. 1.

### Selecting the Preferred Working Projection

A total of five IRs selected their preferred BWP for all 20 IAs using a self-developed simulation software. The software includes a biplane projection simulation module comparable to those integrated in angiography systems, providing surface representations of vessels and aneurysms and allowing an intuitive workflow for batch processing of multiple cases. Collision detection can be deactivated to avoid interference, and the software stores outcome data of selected projections in a structured format for further analysis. In contrast to commercially integrated angiography tools, the self-developed software can be used independently of an angiography suite, thereby enabling standardized evaluation across multiple cases. While a similar study could theoretically be performed with commercial software, this approach ensured flexible, reproducible analysis outside of the clinical workflow. Use of both planes was mandatory. When multiple BWPs were deemed feasible, raters were required to select the most favorable projection. The user interface is shown in supplementary Fig. 1. Volume renderings and X-ray-like forward projections were provided for biplane views, referred to as A and B projections. For the defined purpose of identifying BWPs, the software was configured without the physical restrictions that would exist in a real biplane setting. This way, projections could also be selected where C-arms would collide, either with the patient, or with the table. During the selection process, the orientations of the projections were shown using a surface model of a head (similar to the 3D tools of different vendors), but no reference was made for collisions or other physical constraints. Zooming in on or deleting vessels was not possible. The angle between the corresponding A and B projections of all BWPs was extrapolated. All BWPs were then independently tested for feasibility in a real-world scenario using a model of an ARTIS icono biplane C-arm system (Siemens Healthineers GmbH, Germany) equipped with 30 × 40 cm detectors on both planes.

### Determination of Consent to Working Projections

All 100 selected BWPs were evaluated by five ARs for their suitability for EVT in a randomized and blinded manner, without any additional information. This evaluation was conducted through a binary question that assessed the feasibility of BWPs for intrasaccular treatment. Furthermore, if the suitability was denied, the reason behind this decision was asked. There were three predefined options with the possibility of providing multiple answers: 1) the aneurysm neck was not properly visible, 2) the parent vessel was not properly visible, and 3) the associated vessels were not properly visible. If there was a different reason, this was recorded as a free text (option 4).

For the evaluation, montages of the static biplane projections and a combined horizontal and vertical omnidirectional view in 10° steps around the aneurysm were generated with a starting position of an ordinary view in AP direction, which typically corresponds to a projection of the primary C-arm A. This gave the raters the opportunity to get an impression of the entire anatomical situation in addition to the chosen BWP.

These moving image montages were made available to the raters in random order by use of the VGG Image Annotator 3.0.11 (Visual Geometry Group, Oxford, England). An example of the tool is shown in supplementary Fig. 2.

**Fig. 2:**
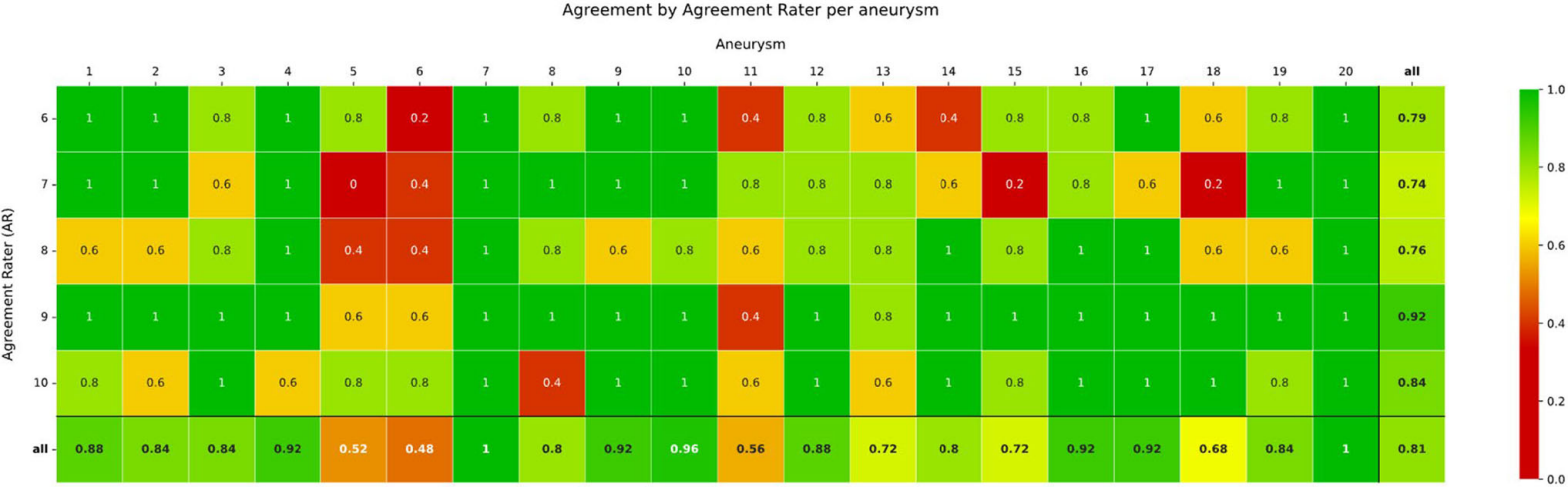
Proportion of agreement of each Agreement Rater for all selected biplane working projections per aneurysm

### Classification of Agreement

The Revised Standards for Interpreting Interrater Agreement Estimates were used. An agreement of 0.91–1 was considered “Very strong agreement,” 0.71–0.90 as “Strong agreement,” 0.51–0.70 as “Moderate agreement,” 0.31–0.50 as “Weak agreement,” and 0.00—0.30 as “Lack of agreement” [15].

### Statistical Analysis

Statistical analysis was performed using Python 3.11 libraries pandas (1.5.3), sklearn (1.5.1), and statsmodel (0.14.2). The aneurysm characteristics were analyzed descriptively. Continuous variables are listed as mean, ± standard deviation (SD), and range. Binary variables are listed as total numbers and percentage. To describe the agreement of two raters, Cohen’s kappa was calculated. For the agreement of more than 2 raters, Fleiss’ kappa was used. Differences between two groups of binary variables were calculated with the χ^2^ test. Depending on the normal distribution, the unpaired t test or the Mann–Whitney U test was used for differences between two groups for continuous variables. Statistical significance was predefined with a *p*-value < 0.05.

## Results

### Determination of Agreement to Working Projections

A Fleiss’ kappa of 0.21 (*p* < 0.001) was obtained across all AR, indicating fair overall agreement. A detailed comparison of individual raters is presented in Table 2.

**Table 2:** Agreement and interrater reliability for chosen projections. AR ID (1) and AR ID (2) indicate the two agreement raters being compared. Each row represents one pairwise comparison among the five agreement raters (6–10)

AR ID (1)	AR ID (2)	Cohen’s kappa	Agreement
6	7	0.36	0.77
6	8	0.23	0.73
6	9	0.34	0.83
6	10	0.11	0.73
7	8	0.25	0.72
7	9	0.19	0.76
7	10	−0.07	0.64
8	9	0.29	0.80
8	10	0.19	0.74
9	10	0.25	0.84

*AR* Agreement rater, *ID* Identification number

The average Cohen’s kappa values decreased within the range of fair agreement. Notably, only rater 10 exhibited a lower level of agreement, with an average Cohen’s kappa of 0.07, reflecting slight agreement.

Considering all selected BWPs per aneurysm, an overall strong agreement of 0.81 was achieved. This agreement varied between the AR, ranging from 0.74 to 0.92. One aneurysm (Aneurysm 6) showed a weak agreement of 0.48 regarding the selected BWP. Three aneurysms had a moderate agreement of 0.52–0.68. For nine aneurysms, there was a strong agreement (0.72–0.88) and for seven aneurysms a very strong agreement (0.92–1) in relation to the selected projections. A detailed breakdown is provided in Fig. 2.

**Fig. 3:**
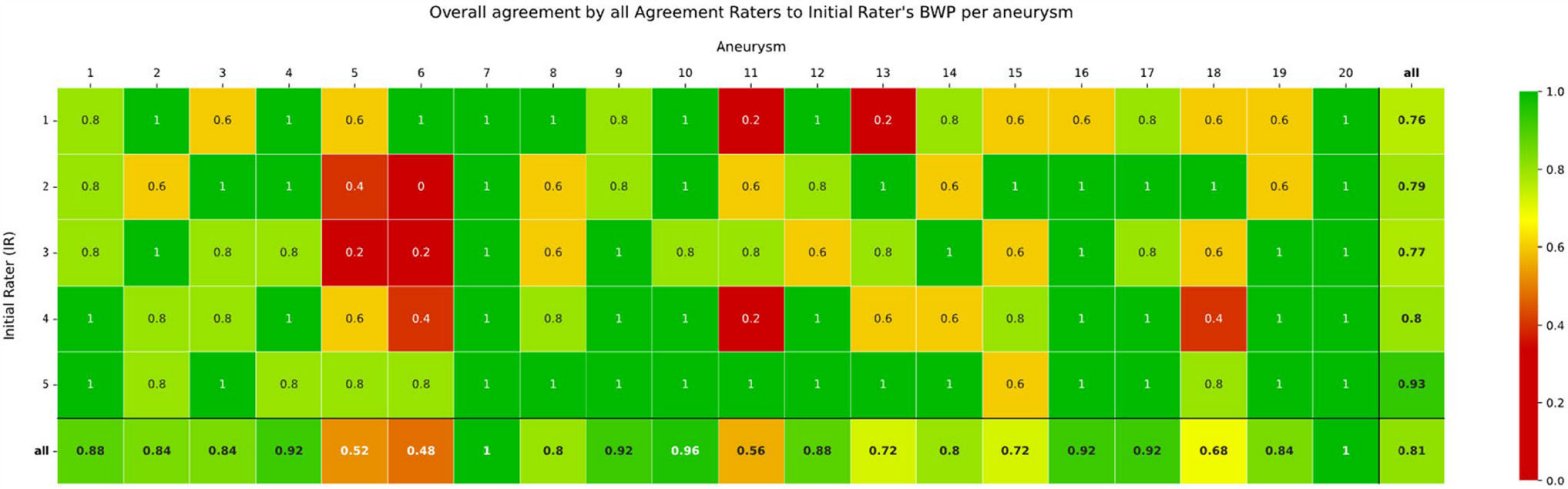
Proportion of agreement to each Initial Rater for all selected biplane working projections per aneurysm

For 19 out of the 20 aneurysms, at least one BWP was available, with all five ARs in agreement. Fifteen had at least two BWPs, nine had at least three BWPs, four had at least four BWPs and two had all five BWPs with a unanimous agreement provided by all ARs. One aneurysm (5) had at least one BWP with almost complete agreement (four out of five ARs) (see Fig. 3). The overall agreement for all BWPs, calculated as Fleiss’ kappa for the entire dataset of five ARs rating 100 projections, was 0.81, varying between 0.76 for the BWPs generated by IR 1 and 0.93 for IR 5. No statistically significant differences in consensus rates were found according to aneurysm size (*p* = 0.57), location (*p* = 0.42), or treatment group (*p* = 0.52).

### Reasons for Rejecting the BWP

Of the 100 BWP, 49 were fully approved by all ARs. In the case of rejection, no clear delineation of the neck, of the parent vessel and of associated vessels were almost evenly distributed, being listed as the reason for BWP rejection 37, 39 and 31 times, respectively. However, reasons other than the previously listed options were only given 20 times (no clear view of the aneurysm in B projection and overlay of other vessels). In 28 of 51 BWPs with at least one rejection, multiple reasons for rejecting the BWP were given. An overview of the reasons for rejection is presented in Table 3 and detailed in supplementary Table 1.

**Table 3:** Distribution of reasons for rejecting a projection in relation to the number of rejecting raters

Total number of ARs that rejected the BWPs	Total number of rejected BWPs	Reasons for rejection				Multiple reasons selected
		Reason 1: NCD Neck	Reason 2: NCD parent vessel	Reason 3: NCD associated vessels	Reason 4: other	
0	49	–	–	–	–	–
1	23	6	9	9	5	4
2	19	17	16	13	6	13
3	3	3	6	2	3	5
4	5	8	6	6	5	4
5	1	3	2	1	1	2
All	100	37	39	31	20	28

*AR* Agreement rater, *BWP* Biplane working projections, *NCD* No clear delineation, *AR* Agreement rater

### Feasibility with Physical Restrictions

**Fig. 4:**
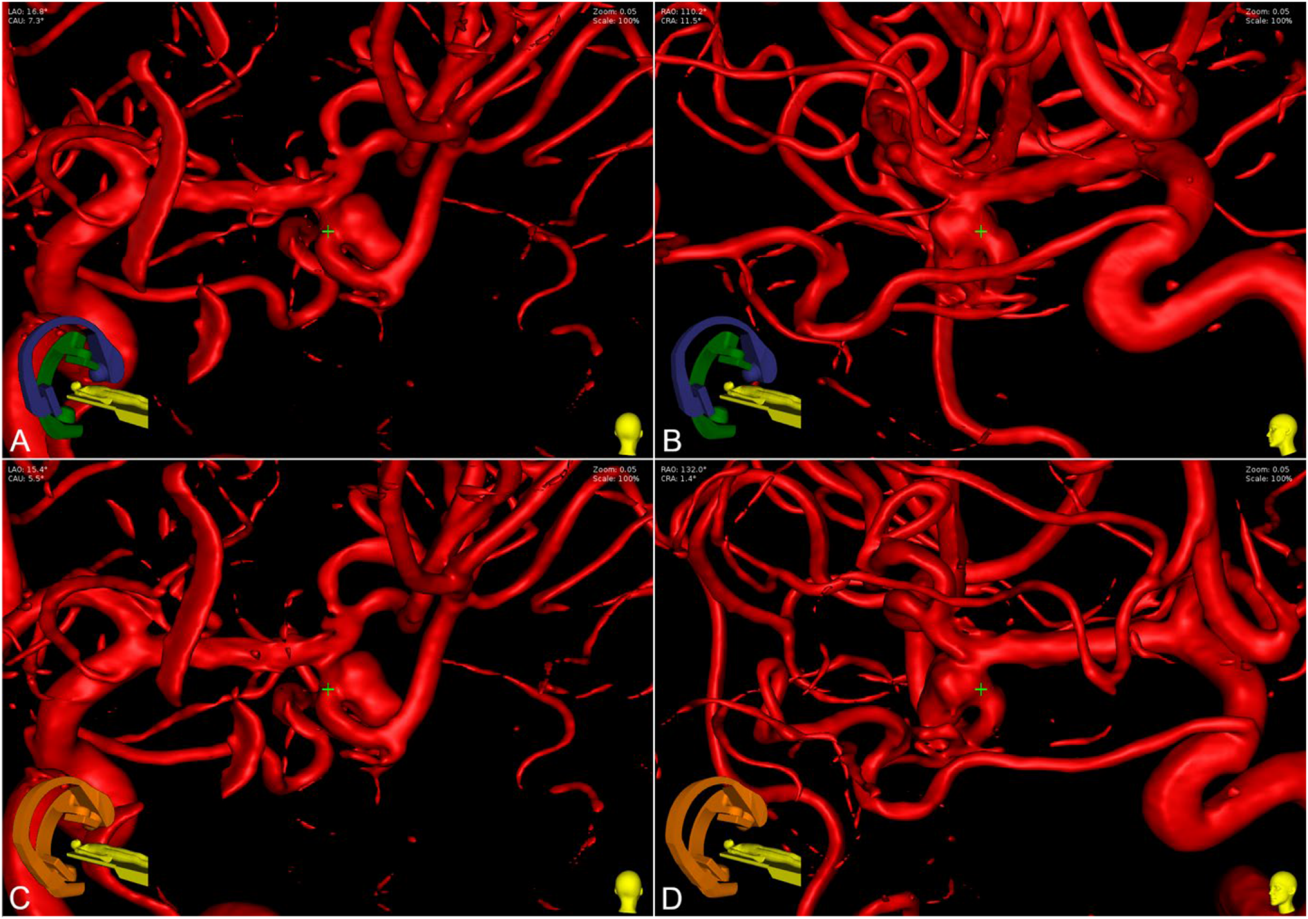
Exemplary case of an anterior communicating artery aneurysm (No. 12). The point of interest (small green cross) is placed at the approximate center of the aneurysm neck. The biplane working projection selected by Initial Rater 2 **A**, **B** was feasible, with no collision between the C-arms (green and blue) or the examination table. In contrast, the projection selected by Initial Rater 5 **C**, **D** was not feasible due to a collision of the two C-arms (orange)

Taking physical restrictions into account, 20% of the total 100 BWPs can be implemented without further modifications, such as by optimizing patient positioning or making minor adjustments to the chosen projection angles. An exemplary case is shown in Fig. 4, and a detailed overview is presented in supplementary Table 2.

## Discussion

Our study demonstrates that despite heterogeneous choices of primarily chosen specific BWP for individual aneurysms, a strong overall agreement was achieved, with a mean κ of 0.81 (range 0.76–0.93) across all raters, as illustrated in Fig. 3. Certain aneurysms (5, 6, 11, 18) exhibited a moderate and weak overall agreement (< 0.7) concerning the selected projections. The main reasons given for these specific aneurysms affected projections were due to the lack of a clear view of the neck (46%), the parent vessel (41%) and the branch vessels (27%).

Kucukay et al. compared the visibility of 122 aneurysms using 2D DSA and 3D DSA with and without VR. Eight standard projections were defined for the 2D DSA. The quality of visibility of the aneurysm neck, aneurysm morphology, and its relationship to the neighboring vessel were assessed in three categories: conclusive, ambiguous, and insufficient. In the 2D DSA, the aneurysm neck was rated as insufficiently visible in 62.3% of cases and ambiguously visible in 2.5%. In contrast, in the 3D DSA with VR, the neck was ambiguously visible in only 2.5% of cases and insufficiently visible in 0%. Similar results were observed regarding the aneurysm’s relationship to the neighboring artery, which was conclusive in only 28.7% of 2D DSA cases but in 96.7% of 3D DSA cases with VR. In our study, projections were rated limiting the option of free rotation, effectively simulating a 2D DSA approach. Compared to the 2D DSA results reported by Kucukay et al., the lack of visibility of the aneurysm neck in our analysis was lower (37% versus 64.8%). Additionally, the rate of a good delineation of the adjunct vessels was higher in our cohort (69% vs 28.7%) [16].

Sugahara et al. also compared 2D DSA and 3D DSA in a study of 36 aneurysms. The 2D DSA was conducted using anterior–posterior and lateral projections. The visibility of the aneurysm neck and the delineation of adjacent vessels were evaluated using a four-point scale (1 = poor, 2 = fair, 3 = good, 4 = excellent). In this study, the visibility of the aneurysm neck in the standard 2D projections was rated, on average, as 2.5 (fair to poor), while the delineation of adjacent vessels received an average score of 2.37[17]. This can primarily be attributed to the fact that in our study, the projection to be assessed was selected in advance for each individual aneurysm, unlike the predetermined standard projections applied to all aneurysms in the studies mentioned above. These findings highlight the limitations of relying on standard projections for the EVT of aneurysms and highlights the importance of a clearly defined working projection.

There is also a noticeable difference among individual IRs. While four of the IRs received overall agreement rates of 0.76–0.80 (strong agreement) for their selected projections, the agreement for IR 5’s chosen projections was significantly higher at 0.93 (very strong agreement). This indicates that even for seemingly challenging aneurysms, a feasible working projection can be found. For example, the agreement on the BWP for aneurysm No. 5 was 60% for IRs 1 and 4, 40% for IR 2, and only 20% for IR 3. However, the BWP of IR 5 received 80% agreement from the ARs. Similarly, for aneurysm No. 6 (Fig. 3), the agreement for the chosen projections of IRs 2–4 ranged from 0 to 40%, whereas IR 5 selected a projection with 80% agreement, and IR 1 identified a projection that received 100% agreement. It is conceivable that some raters consistently select projections that align more closely with general consensus, which may provide useful insights for training and the establishment of best practices. Conversely, some aneurysms, such as aneurysm No. 20, achieved 100% overall agreement despite differing projections from various IRs. This underlines the heterogeneity regarding the chosen BWP based on the aneurysm, as well as individual differences depending on the INR. Each aneurysm is a unique case, and there is no single projection that is universally optimal for all aneurysms. This is evident from our results, which show a wide range of selected projections for aneurysms located in the same region.

More important than a “one projection fits all” approach is identifying characteristics that enable an aneurysm specific working projection for safe endovascular treatments. In our data, besides the lack of a clear view of the neck (37%) and associated vessels (31%), the primary reason for rejecting a working projection was the absence of a clear view of the parent vessel (39%). There is a general consensus that a good working projection should avoid obscuring the aneurysm neck, the parent vessel, or the branching vessels. However, robust data demonstrating the impact of these criteria on feasibility and treatment risk are lacking. Complication rates for coil embolization is reported between 6.7% and 17.4%, depending on aneurysm characteristics and treatment strategy [18–21]. However, only in a minority of cases, a clear cause is reported. It is conceivable that some complications arise from suboptimal BWP, for example due to insufficient depiction of the aneurysm neck, the parent vessel, or overlapping vessels. Our finding that at least one projection per aneurysm achieved broad agreement suggests that consensus projections could serve as valuable references for clinical practice. Similar to the use of pre-procedural simulation for device sizing, which has been shown to reduce procedure times and the number of devices required, such projections may facilitate more efficient procedure planning. For example, identifying an optimal projection in advance could support improved patient head positioning and the appropriate selection of treatment techniques [22, 23]. Consensus projections may also provide benchmarks for less experienced operators and enhance training by highlighting views that incorporate the aforementioned criteria. Integrating such projections into practice could therefore improve both the safety and reproducibility of endosaccular aneurysm treatment.

### Outlook

20% of the selected BWP can be utilized as working projections without modifications, however, 80% face physical restrictions, such as collisions between the C-arm and the patient or table. By adjusting the patient’s head position or making slight angle deviations, the proportion of utilizable projections could increase. The extent to which these changes affect the usability of the selected BWP needs to be investigated in further studies. Notably, our data provide a basis for the development of future artificial intelligence-assisted applications that could support the optimization of biplane working projections and facilitate the translation of idealized views into clinical practice.

### Limitations

This study focused exclusively on relatively small, narrow-neck aneurysms of the anterior circulation. Therefore, the results may not be generalizable to giant or fusiform aneurysms, aneurysms of the posterior circulation, or to cases requiring adjunctive devices such as stents or flow diverters. The consensus reported in this study refers to idealized projections without physical constraints. In real-world practice, compromises may be necessary due to C-arm collisions, patient positioning, or anatomical restrictions. The retrospective data collection may result in selection bias. However, we mitigated this by randomly selecting study subjects. Additionally, the determination of the chosen BWP could be biased due to the presence of overlapping vessels in the corresponding real-world 2D run. To minimize this effect, zooming in or deselecting and hiding vessels was not permitted.

## Conclusion

The BWP selected by each IR is heterogeneous. Despite this, there is an overall positive consensus regarding the identification of appropriate BWP. Important factors for evaluating a good BWP include a clear visualization of the aneurysm neck, parent vessels, and branching arteries. However, it should be emphasized that a high consensus on an optimal view does not necessarily mean that this view is readily attainable in the angiography suite. Further research is required to explore the possible benefit of adjusting the patient’s head position or making slight angle deviations in BWPs with physical restrictions and furthermore, to evaluate the risks associated with suboptimal BWPs in EVT.
